# Decennial Ward‐Level Influence of Demographic, Farming, and Economic Predictors on All‐Cause Mortality

**DOI:** 10.1111/ajr.70016

**Published:** 2025-02-24

**Authors:** Kelly Trearty, Brendan Bunting, John Mallett

**Affiliations:** ^1^ Department of Psychology Ulster University—Derry Londonderry Campus Derry UK

**Keywords:** aggregated ward data, farming intensity scores, mortality patterns, NINIS data, regression analysis

## Abstract

**Objective:**

This study has arisen in response to a lack of studies examining how farming affects mortality patterns across areas of Northern Ireland (NI) over a 10‐year period. This paper aims to investigate whether agriculturally intensive electoral Wards have higher mortality rates compared to non‐agriculturally based Wards, controlling for relevant factors.

**Methods:**

The population census and farm census information was downloaded from the Northern Ireland Neighbourhood Service (NINIS) website to construct two original mortality‐based datasets. Linear regression was used for the analysis.

**Design:**

Decennial Ward‐Level Influence of Demographic, Farming, and Economic Predictors on All‐Cause Mortality.

**Setting:**

Five hundred and eighty‐two Ward areas of Northern Ireland.

**Main Outcome Measure:**

Mortality risk within Ward areas.

**Results:**

Findings showed larger amounts of natural log of the population, 65 to 100+ year‐olds, limiting long‐term illnesses, Farming Intensity Scores, residents living alone and full‐time workers within Wards were predictive of mortality risk within those Wards.

**Conclusions:**

This study is the first of its kind in NI to provide evidence for Farming Intensity Scores explaining the variation of mortality rates between areas, in addition to many of the usual predictors.

**Summary**

- What is already known on this subject
  ○ Farmers have the highest mortality risk worldwide compared with other occupations.
  ○ Death rates were substantially higher in countries with larger proportions of: 65+ year‐olds, lower education (25+ years old), near‐poverty incomes, and farming occupations/activity.
  ○ This study is the first of its kind to provide evidence for the role of Farming Intensity Scores and related area‐level predictors as explanatory measures for area‐level mortality rates.
- What this paper adds
  ○ Population and Farm Census data were used to compile two mortality‐based datasets for the 2001 and 2011 population decennial periods in Northern Ireland.
  ○ Farms, farmers, livestock, and arable variables were measured using a latent construct of *Farming Intensity*.
  ○ Agricultural Wards had higher mortality rates compared with non‐farming Wards in Northern Ireland, and potential explanatory factors are described.

## 1 Introduction

Farmers have the highest mortality risk worldwide compared against other occupations [1]. In 2019, the Northern Ireland Statistics and Research Agency (NISRA) reported approximately 75% of Northern Ireland's (NI's) landscape was utilised for agricultural purposes [2]. On a daily basis, farmers encounter an abundance of health and safety hazards, resulting in fatal and non‐fatal farm accidents increasing annually [3, 4]. Therefore, this study's main research question hinges on whether demographic, farming, and economic predictors help resolve some of the unexplained variation of all‐cause mortality risk in NI Wards (2001; 2011).

Young and colleagues [5] investigated self‐rated health indicators on mortality patterns between counties of the United Kingdom, using data from census‐based longitudinal studies available for England, Scotland, Wales, and NI. NI results indicated individuals with higher levels of disadvantage were 5.4 times more likely to report poor health, and those with poor health were 2.7 times more likely to die than those reporting good health. Smyth and colleagues [1] investigated mortality trends of Irish farmers (15–64 years) against other occupational groups (2000–2006). Results demonstrated that agricultural workers (7.4 times) and farmers (5.1 times) recorded the highest all‐cause mortality risk compared to other salaried employees (e.g. agents, contractors, insurance brokers, sales representatives, etc.). Chan and associates [6] investigated sociodemographics on mortality rates in 458 countries with 100,000+ population (1998–2002). In line with previous research [5], results indicated death rates were substantially higher in countries with larger proportions of: 65+ year‐olds, lower education (25+ years old), near‐poverty incomes, and farming occupations/activity.

Haan and colleagues [7] investigated the association between socio‐economic status (SES) and all‐cause mortality risk of 35+ year olds (*n* = 1811) within high poverty areas (over 9‐years) using the Alameda County Study, California. They used *poverty area* to measure an area's low SES, derived from an individual's social and physical environment. Findings indicated that the populace of poverty areas had a RR 1.7 [1.2–2.44] times increased risk of death compared to non‐poverty areas. Building on this research, Yen and Kaplan [8] investigated neighbourhood social environments on the 11‐year risk of mortality (*n* = 1129) using census and area‐level data from the Alameda County Study, California. Results matched those of Haan and collaborators [7] indicating deprived social environments within neighbourhoods in 1983 had a RR 1.6 [1.15–2.18] times increased risk of mortality by 1994.

Earlier research has advocated for more data linkages across national population censuses and agricultural censuses at small area level to enhance the overall usefulness of such data within research [9]. Therefore, this study utilised the latest census information available (2001; 2011) from the Northern Ireland Neighbourhood Information Service (NINIS) to examine the links between farming activity and demographics on mortality risk (NISRA 2019). In addition, the current study expanded the outcome [10] and scope of predictors [11] utilised within previous research to reduce the amount of unexplained variation related to mortality risk which remains between small areas of NI. The current study was designed in response to the limited number of databases available [12] to investigate the wider socio‐ecological [13] predictors of mortality risk within NI Wards [14].

Building on previous research, this study makes use of a novel composite measure of *Farming Intensity* to assess agricultural activity at electoral Ward level; based on aggregated numbers of farms, cattle, pigs, and poultry, alongside the proportion of farmers and agricultural grassland [15]. In addition, farming is a resource‐dependent occupation where the health and well‐being of the farmer directly impact their ability to maintain an adequate income from agricultural production on their farm business. Previous research has indicated *Gross Margin* is the most commonly used indicator of agricultural economic size and farming capital within areas [16]. Therefore, *Average Standard Gross Margin* was utilised as a measure of *farming profit* at Ward level so that inferences could be made relating to an area's agricultural deprivation/affluence on mortality risk.

### 1.1 Research Hypotheses

This study hypothesised that farms, farmers, livestock, and arable variables are measuring the same underlying factor of *Farming Intensity* in 2001 and 2011. This composite *Farming Intensity* variable will be estimated and used to predict Ward‐level mortality risk. Holding all other covariates constant, the current study hypothesised that Wards with larger amounts of *natural log of the population, 65 to 100+ year‐olds, males, residents living alone, below degree qualifications, full‐time workers, limiting long‐term illnesses (LLTIs), unpaid carers* and *Farming Intensity Scores* in a Ward will increase the risk of *death* within those Wards. Keeping all variables equal, this study hypothesised that Wards with higher *multiple deprivation* rankings (or least deprived Wards) and greater *farming profit* (or agricultural affluence) will decrease the risk of *mortality* within those Wards.

## 2 Methodology

### 2.1 Context of Study

Northern Ireland Neighbourhood Information Service (NINIS) is a web‐based service that holds nationally representative Population Census and Farm Census information within its open data portal [2]. This data was publicly available, and ethical approval was granted by the University Ethics Committee (2019). For this study, the two most recent population censuses available were utilised (2001; 2011) and the farm census data from the same years were compiled. The database set‐up emulated that of Luke and Krauss [17], converting all area‐level variables into the proportion of their summed total. The 582 Wards of NI contained a wide range of population sizes, ranging from 760 to 9500 residents. This study emulated the methodology of McGranahan [18] who rescaled population size at Ward level by converting it to a *natural logarithm of the population score* for each Ward and then used this variable as a covariate in their regression model.

### 2.2 Measures

All variables were extracted as counts at ward level taken from the 2001 and 2011 Censuses (Appendix S1) and different transformations were performed to operationalise their usefulness (Appendices S2 and S3) [12].

#### 2.2.1 Deaths

The outcome variable was the *total number of deaths* recorded over a 12‐month period. The causes of death included in this variable were: malignant neoplasms C00‐C97 (e.g. cancers, etc.); circulatory diseases I00‐I97 (e.g. heart disease, stroke, blood clots, etc.); respiratory diseases J00–J99 (e.g. pneumonia, asthma, bronchitis, influenza, etc.); and external causes V01–Y98, X60–X84, Y10–Y34, Y87.0, Y87.2 (e.g. road traffic accidents, falls, suicide, undetermined intent, fires, poisoning, assault etc.). The death variable for each ward remained in its raw form as a count.

*Ward Population* was the total number of people living in each Ward, and its *natural log* was calculated.

*Age* was recorded as an individual's age on their last birthday before Census day. The age variable was operationalised as the proportion of people in each Ward aged *65 to 100+ years old*.

*Gender* was likewise operationalised from individual Census data as the proportion of *males* in each Ward.

*Marital Status* represented a person's legal marital status on Census day. It was operationalised as the proportion of Ward residents aged 16+ *living alone* (summed single, separated, divorced, and widowed).

*Qualifications* represented the highest level of education obtained by Census day. It was again recorded as the proportion of Ward residents aged 16 and over with *below degree* level qualifications (summed no qualifications, NVQ Levels 1–3, apprenticeships, and others).

*Limiting Long‐Term Illness (LLTI)* represented a combination of six physical and five mental health conditions. This variable was coded as the proportion of Ward residents who reported *having an LLTI* or disability lasting, or expected to last, a minimum of 12 months from Census day.

*Unpaid Carers* was coded as the proportion of residents aged 16+ who were providing any *unpaid care (1+ h)* weekly.

*Hours Worked Weekly* was recorded as the proportion of Ward residents aged 16–74 years *working full‐time (31+ h)* weekly in their main job, the week before the Census enumeration, including paid and unpaid overtime.

*Northern Ireland Multiple Deprivation Measure (NIMDM)* reflected the overall weighted score across seven deprivation domains; 2005 and 2010 NIMDM variables were utilised within datasets. *NIMDM* ranked areas from most deprived (1) to least deprived (582) Wards.

*Average Standard Gross Margin (SGM)* represented the annual farming profit earned from arable and/or livestock production per Ward. In 2011, Average SGM changed to the Average Standard Output (SO) within the Farm Census, which calculated the average number of on‐farm hours worked annually per Ward [19]. In line with previous research [16], an approximate equivalence was assumed by including the 2001 and 2010 *SGM* information within datasets as a proxy for farm profit where one unit equals €1000 [2]. SGM (or farm profit) was calculated using the farm business revenue minus costs of farm activities equating to profits at Ward level. The 2001 and 2010 *farm profit* variables remained unchanged in their count form per Ward.

*Farming Intensity* was computed as a composite factor score for each Ward using six indicator items from the Farm Census [19]. *Farming Intensity* represented the proportions of farmers and grass‐based farming per Ward, alongside the total number of farms, cattle, pigs, and poultry per Ward.

*Farms* represented the total number of active agricultural holdings per Ward from the Farm Census. Active farms were defined as spatial units encapsulating 1+ hectares of farmland used for crops and/or livestock production. *Farms* remained as a count per Ward.

*Agricultural Labour* represented the total number of active farmers (16+ years old) employed within the agricultural industry for 20 (39%) or more weeks per year. This study merged farmers, spouses, and other farm workers to create the summed total of *agricultural labourers* (hereafter referred to as *farmers*) per Ward from the Farm Census. The total number of *farmers* was converted into its proportion relative to the total population over 16 years old per Ward.

*Grass* represented the total grass‐based farming in hectares per Ward, excluding rough grazing, farm woodlands, and non‐agricultural land in the Farm Census. It was converted into the proportion of *grass* in hectares relative to the *total hectare area farmed* per Ward.

*Cattle* represented the total number of cattle in each Ward from the Farm Census.

*Pigs* represented the total number of pigs per Ward from the Farm Census.

*Poultry* represented the total number of poultry in each Ward from the Farm Census.

The livestock variables (number of *cattle, pigs*, and *poultry* within each Ward) were rescaled from large continuous variables into four‐level categorical variables (Scored 0–3). Wards holding a total of 0–3 of any livestock were recoded as 0 to represent *none of that animal* within that Ward. The remaining amount of each livestock variable was divided by three to create equally banded categories across Wards, representing small (1), medium (2), and large (3) quantities of that animal per Ward.

### 2.3 Statistical Analysis

The outcome variable (number of *deaths* per Ward) was treated as continuous within a linear regression analysis. Other variables had extremely large values. Therefore, *farms, grass, living alone*, and *farming profit* variables were rescaled by dividing them by 10, and *deprivation* ranks were divided by 100 in order to rescale their large variances [20]. Standardised results were reported throughout this paper for the purpose of comparison. Mplus 8.1 was used to conduct the initial confirmatory factor analysis (CFA) of farming‐related variables [21]. Maximum likelihood with robust standard errors (MLR) was performed, as the model contained both continuous and categorical indicators. MLR also provided unbiased estimates by efficiently handling non‐normally distributed data. Factor scores were computed for *Farming Intensity* using the *refined regression* method [22] and were saved for use as a predictor of mortality in the subsequent multiple linear regression.

## 3 Results

### 3.1 Factor Model

The 2001 goodness of fit result was: *x* ^2^ (6, *N* = 582) = 29.371, *p* < 0001, CFI = 0.987, RMSEA = 0.082 (90% CI [0.054–0.112]), SRMR = 0.018. In 2011, the goodness of fit result was: *x* ^2^ (6, *N* = 582) = 8.578, *p* > 0.05, CFI = 0.998, RMSEA = 0.027 (90% CI [0.000–0.065]), SRMR = 0.011. CFA results indicated the *Farming Intensity* model (Table 1) comprising *farms, farmers, grass, cattle, pigs* and *poultry* accompanied by three correlated residuals can be accepted in 2001 and 2011. Estimated factor loadings were similar and above 0.7 for 2001 and 2011.

**TABLE 1:** Standardised CFA results of Farming Intensity in 2001 and 2011.

Farming Intensity	2001				2011			
	Loading	S.E.	*z*	*p* value	Loading	S.E.	*z*	*p* value
Indicators								
Cattle	0.967	0.008	125.634	**0.000**	0.964	0.010	96.853	**0.000**
Farmers	0.888	0.013	69.922	**0.000**	0.897	0.013	70.259	**0.000**
Farms	0.871	0.011	78.469	**0.000**	0.885	0.014	64.678	**0.000**
Poultry	0.877	0.013	68.388	**0.000**	0.782	0.023	34.144	**0.000**
Grass	0.748	0.020	36.805	**0.000**	0.756	0.020	38.200	**0.000**
Pigs	0.709	0.025	28.015	**0.000**	0.699	0.028	24.581	**0.000**
Residual Correlations								
Farms WITH Farmers	0.759	0.037	20.487	**0.000**	0.712	0.054	13.225	**0.006**
Grass WITH Cattle	0.734	0.045	16.303	**0.000**	0.807	0.042	19.050	**0.000**
Farms WITH Cattle	0.158	0.043	3.642	**0.000**	0.151	0.055	2.722	**0.000**

*Note: p* values in bold represent *p* < 0.05 statistical significance.

### 3.2 Regression Model

Descriptive statistics for each variable are available within the Supporting Information. Linear regression was used to examine the unique effects of all 11 covariates on deaths within Wards (*n* = 582) both years (Table 2). Holding all other predictor variables constant, results indicated that a 1‐unit change in the *natural log of the population* resulted in an increase in the *death* rates in these Wards for both years. Keeping all other predictors equal, results indicated that a 1% change in *65 to 100+‐year‐olds* resulted in an increase in the *mortality* rates in these Wards for both years. Holding all other predictor variables constant, results indicated that a 1% change in affirmative *LLTIs* resulted in an increase in the *death* rates in these Wards for both years. Keeping all other predictors equal, results indicated that a 1‐unit change in *Farming Intensity Scores* resulted in an increase in the *mortality* rates in these Wards for both years.

**TABLE 2:** Standardised regression results for Ward‐level demographics, farming, and economic predictors on death in 2001 and 2011.

Covariates	2001				2011			
	*β*	S.E.	*z*	*p* value	*β*	S.E.	*z*	*p* value
Natural log of the population	0.651	0.030	21.452	**0.000**	0.704	0.034	20.430	**0.000**
Aged 65 to 100+	0.370	0.044	8.455	**0.000**	0.404	0.050	8.026	**0.000**
LLTI	0.379	0.067	5.655	**0.000**	0.356	0.071	5.030	**0.000**
Farming intensity scores	0.097	0.041	2.400	**0.016**	0.090	0.041	2.195	**0.028**
Living alone	0.033	0.054	0.620	0.535	0.248	0.054	4.555	**0.000**
Full‐time hours	0.015	0.033	0.461	0.645	0.084	0.027	3.042	**0.002**
Below degree	−0.169	0.041	−4.094	**0.000**	−0.152	0.046	−3.331	**0.001**
Unpaid carers	−0.056	0.035	−1.581	0.114	−0.051	0.033	−1.541	0.123
Farming profit	−0.018	0.030	−0.598	0.550	−0.027	0.030	−0.881	0.378
Multiple deprivation	−0.011	0.058	−0.191	0.849	0.107	0.065	1.656	0.098
Males	−0.009	0.040	−0.232	0.816	−0.028	0.034	−0.824	0.410
***Model R‐squared***	0.660	0.023	28.855	**0.000**	0.662	0.023	29.082	**0.000**

*Note: p* values in bold represent *p* < 0.05 statistical significance.

Holding all other predictor variables constant, results indicated that a 1% change in *living alone* resulted in an increase in the *death* rates in 2011 Wards. Keeping all other predictors equal, results indicated that a 1% change in *full‐time workers* resulted in an increase in the *mortality* rates within 2011 Wards. Holding all other predictor variables constant, results indicated that a 1% change in *below degree* qualifications resulted in a decrease in the *death* rates in these Wards both years. Keeping all other predictors equal, results indicated that a 1% change in *unpaid carers* resulted in a decrease in the *death* rates in these Wards; but results may have occurred by chance both years.

Holding all other predictor variables constant, results indicated that a 1‐unit change in *farming profit* resulted in a decrease in the *death* rates in these Wards; but results may have occurred by chance both years. Keeping all other predictors equal, results indicated that a 1 rank change in *deprivation* resulted in a decrease (2001) and an increase (2011) in the *death* rates in these Wards; but results may have occurred by chance both years. Holding all other predictor variables constant, results indicated that a 1% change in *males* resulted in a decrease in the *death* rates in these Wards; but results may have occurred by chance both years. The r‐square for models 2001 and 2011 was the same (*R* ^2^ = 0.660 and 0.662, respectively).

## 4 Discussion

### 4.1 Factor Model

CFA results yielded reasonable model fit for 2001 and 2011 datasets; furthermore, all factor loadings and correlated residuals of the six items were statistically significant. *Cattle* were the strongest indicator of *Farming Intensity* in both years, emphasising the importance of livestock farming within agriculturally saturated Wards. *Farmers* and *farms* were the second strongest indicators of *Farming Intensity* in both years, emphasising the importance of farmland and labour efficiency within the model. Therefore, CFA results supported the *Farming Intensity* hypothesis, as the model (farms, farmers, grass, cattle, pigs, poultry) measured the same underlying construct in 2001 and 2011 Wards consistently.

### 4.2 Regression Model

As expected, current results showed that the *natural log of the population* was the strongest predictor of death risk in both years (approximately twice the predictor of death risk of age). However, it was calculated from the *Ward population* variable; therefore, its strength can be ignored as the findings were simply the function of risk exposure each Ward had to mortality [18]. Current results indicated *65 to 100+‐year‐olds* were the real strongest predictor of death risk in Wards in both years. As hypothesised, these results match those observed in earlier studies and provide evidence that areas with larger amounts of residents *aged 65 to 100+ years old* have a higher risk of death compared to areas with more occupants aged *under 64 years old* [5, 6]. Current findings are supported by NI national statistics reporting that a majority of deaths occurred within those over 65 years old in 2001 (87%) and 2011 (80%) [2].

LLTI was the second strongest predictor of death risk in Wards in both years. As hypothesised, current results provide evidence that areas with larger amounts of inhabitants experiencing *LLTIs* have a higher risk of mortality in both years compared to areas with more dwellers reporting *no LLTIs*. These findings are in line with previous research concluding that areas with larger amounts of affirmative LLTIs have three times the higher risk of mortality [5]. Current results showed *Farming Intensity Scores* were the third strongest predictor of mortality risk in Wards in both years. As hypothesised, results provide evidence that areas with larger amounts of *agricultural activity* have a higher risk of death in both years compared to areas with greater scores in *no agricultural activity*.

Current results demonstrated that the physical environment (farming intensification) of Wards has a significant influence on the interaction between lower SES areas and increased death risk [1, 6, 7, 23]. Current results agree with Yen and Kaplan [8], who concluded that those living in low SES areas had a 58% increased likelihood of dying compared to those living in high SES areas. Likewise, the simultaneous decrease in farmers, farms, and mortality rates longitudinally supports the need for the current research [2]. Previous research has provided plausible explanations for this pattern of findings, concluding that Ward‐level agricultural work operates additively to premature death risk via lower status work, job stressors, nonstandard employment, workplace hazard exposures, and workplace contextual factors [24].

Current results indicated *living alone* was a non‐significant predictor of death in 2001 Wards and a statistically significant predictor of death in 2011 Wards. As hypothesised, current 2011 findings are in line with those of previous research and provide evidence that areas with larger amounts of people *living alone* have a higher risk of mortality compared to areas with more dwellers *cohabiting*. Current 2011 results are supported by earlier research concluding that *living alone* was a strong psychosocial risk factor of mortality as it creates a sense of isolation [8], while its secondary effects (e.g. poor health behaviours, etc.) increase the risk of LLTIs (which are also strong risk factors of mortality) [25]. Current 2001 results disagree with those obtained in previous research; this may have occurred because in 2001 there were 629 128 residents *living alone* (single, separated, divorced, widowed). However, by 2011 the total number of people *living alone* increased by 20% (*n* = 749 466) across Wards [2].

Current results showed the *full‐time worker* variable was a non‐significant predictor of death risk in 2001 Wards and a statistically significant predictor of death risk in 2011 Wards. As hypothesised, 2011 results match those observed in earlier studies and provide evidence that areas with larger amounts of residents *working full‐time* (31+ h) weekly have a higher risk of death compared to areas with more occupants *working part‐time* (1–30 h) weekly. Consistent with the findings of Wong et al. [26], a plausible explanation for the 2011 results is that unsociable hours may result in a poor work‐life balance, increasing the risk of morbidities (e.g. chronic fatigue; injury; poor physical/mental health, etc.) and mortality.

Current results indicated that *below degree* qualifications were a statistically significant protector against mortality risk in Wards in both years. Surprisingly, current findings showed that areas with larger amounts of inhabitants with *above degree* qualifications have a higher risk of mortality compared to areas with more dwellers reporting *below degree* qualifications. *Below degree* results failed to support the hypothesis in both years as findings were the reverse of what was hypothesised and contradicted previous research [27]. Current results diverge from those obtained in previous research presumably due to: (i) *above degree* qualifications increasing by 2% between 2001 and 2011; and (ii) approximately 65% of the NI population living in the 54% of Wards classified as urban areas [2] and this high urban population density may have skewed the direction of the results. In addition, one could infer that low income farms forced some farmers to obtain off‐farm work (requiring an *above degree* qualification), resulting in farmers working simultaneously on the farm and in their off‐farm positions, which increased the *above degree* risk of mortality [26].

Current results indicated *unpaid carers* was a non‐significant protector against mortality in Wards both years. Unexpectedly, these results indicated areas with larger amounts of *no unpaid carers* have a higher risk of mortality compared to areas with more dwellers providing 1+ h of *unpaid care*; but findings were not generalizable to the population. *Unpaid carers* failed to support its hypothesis both years, as results were the reverse of what was hypothesised [28]. Despite being non‐significant, the direction of these results dovetail with those obtained by other researchers concluding that caregiving was associated with reduced mortality risk [29]. Current results may have occurred as approximately 35% of the population live in rural areas where the impact of caregiving on mortality risk may differ by societal contexts (farming households generally provide inter‐generational unpaid care) [2].

Current results showed *farming profit* was a non‐significant protector against mortality in Wards both years. These results indicated areas with larger amounts of *farming profit* have a decreased risk of death compared to areas with lower amounts of *farming profit*. Like Yen and Kaplan [8] these findings demonstrate Ward‐level income contributes to an area's risk of death, but results may have occurred by chance and failed to support its hypothesis both years. Current non‐significant results indicated areas ranked *least deprived* (582) had a lower risk of death (protective) in 2001 and had a greater risk of death (predictive) in 2011 compared to areas ranked the *most deprived* (1) both years. Despite 2011 results being reasonable theoretically, *deprivation* failed to support its hypothesis both years, as results may have occurred by chance and were not generalisable to the population. Current results contradict (2001) and support (2011) previous research concluding that increasing levels of disadvantage were associated with an increased risk of mortality at area level [30].

Current results showed *males* were a non‐significant protector against mortality in Wards both years. Current results contradicted previous research [7] showing Wards with larger amounts of *female* populace had a higher risk of mortality compared to areas with larger *male* dwellers. Therefore, *males* failed to support their hypothesis both years as results were the reverse of what was hypothesised and may have occurred by chance. These results may have occurred due to higher female deaths observed in both years; 2001 reported 7506 female deaths (male = 7007) and 2011 reported 7286 female deaths (male = 6918) [2].

### 4.3 Future Direction

Building on previous research, future researchers should investigate (2001, 2011, 2022) if the relationship between exogenous variables (natural log of the population; below degree) to death may be strengthened if intercepted by three mediating variables (LLTI's, full‐time hours, unpaid carers) within Wards both years. Likewise, future researchers should investigate if the relationship between exogenous variables (65+ year olds, males, living alone, deprivation, farming profits) to death may be strengthened if intercepted by four mediating variables (Farming Intensity Scores, LLTI's, full‐time hours, unpaid carers) within Wards in both years. Multiple group analysis should be used to cross‐validate these cause‐and‐effect relationships relating to death between Census years [14].

### 4.4 Strengths and Weaknesses

As a result of this study analysing aggregated data, researchers must be cautious not to make an *ecological fallacy* during the interpretation of findings [31]. A lesser known feature of NINIS data was its ability to create new datasets at Ward level [2] linking multi‐source information using geographical indicators to conduct new neighbourhood effects research across NI. Building two original datasets was free, fast, and it rebuilt the current information's utility [32]. As NINIS data was spatially aggregated, a small degree of information loss exists due to NISRA aggregation procedures [2].

This study applied an interdisciplinary approach to push health science outside its discipline to address new transdisciplinary research questions (agro‐ecological science). These new datasets have the resources to detect health inequalities and mortality patterns that may otherwise remain undiscovered. Ward‐level data was appropriate for this study as individual‐level data cannot fully portray neighbourhood social environmental dimensions [33]. NINIS aggregated area‐level data provide benefits of efficiency and power. Conclusions drawn from these multiple data sources are firmer and reduce the risk of error, in contrast to those drawn from only a single source.

## 5 Conclusions

This study is based on a socio‐ecological model which addresses, amongst others, agro‐ecological questions especially in relation to area‐level Farming Intensity. This study's results (replicated on two occasions) improve our understanding of the key drivers of Ward‐level mortality risk using available decennial data [12]. Current results provide evidence in 2001 and 2011 that the natural log of the population, 65 to 100+ year olds, LLTIs, and Farming Intensity Scores directly account for some of the variation in deaths between Wards of NI. Results provide evidence in 2011 that living alone and full‐time hours directly account for some of the variation in deaths between Wards of NI. In line with earlier area‐level research, results support the aggregated interpretation that higher levels of farming activity within a Ward increase the risk of mortality within those Wards. In conclusion, this research is the first of its kind in NI to provide additional support for the use of aggregated area‐level data in explaining variation of mortality rates between areas.

The current study provides strong evidence that the linkage of population censuses with agricultural censuses from the NINIS data warehouse has a variety of benefits for NI [12], more than just being able to release nationally representative summary statistics earlier; it also facilitates a wide range of research purposes. This study will encourage future researchers to merge the newest 2022 census information (once it is available online) with the current datasets to facilitate new 2022 cross‐sectional and longitudinal research questions (2001–2011‐2022) within NI wards. Future investigators could use this paper's methodology of Farming Intensity Scores as a contextual mechanism to investigate whether agriculturally saturated wards have a higher mortality risk than non‐agriculturally wards of NI (2001; 2011; 2022). Moreover, inter‐disciplinary researchers could replicate these datasets for a comparative analyses at a small area level between countries (using NI, England, Scotland, Wales, or any European country that collects agricultural census information). Results from this research would allow identification of agricultural areas with higher risk mortality and tailoring of educational strategies in targeted areas to help reduce farmers' injuries and death.

## Author Contributions

**Kelly Trearty:** conceptualization, investigation, writing – original draft, writing – review and editing, visualization, validation, methodology, software, formal analysis, project administration, data curation, resources. **Brendan Bunting:** data curation, supervision, resources, project administration, formal analysis, software, methodology, validation, visualization, writing – review and editing, writing – original draft, investigation, conceptualization. **John Mallett:** data curation, supervision, resources, project administration, formal analysis, software, methodology, validation, visualization, writing – review and editing, writing – original draft, investigation, conceptualization.

## Conflicts of Interest

The authors declare no conflicts of interest.

## Supporting information


Appendix S1.

## Data Availability

This data were publicly available and ethical approval was granted by Ulster University Research Ethics Committee in January 2019 (Reference ID: B00573168).

## References

[1] B. Smyth , D. S. Evans , A. Kelly , L. Cullen , and D. O' Donovan , “The Farming Population in Ireland: Mortality Trends During the Celtic Tiger Years,” European Journal of Public Health 23, no. 1 (2012): 50–55. 22436692 10.1093/eurpub/cks017

[2] NISRA , “NINIS Northern Ireland Neighbourhood Information Service. (Northern Ireland Statistics and Research Agency), 2019”, https://www.ninis2.nisra.gov.uk/public/Home.aspx.

[3] D. Cole , Understanding the Links Between Agriculture and Health: Occupational Health Hazards of Agriculture (International Food Policy Ressearch Institute, 2006).

[4] M. J. Lee , D. T. Cawley , J. P. Ng , and K. Kaar , “Trends in the Fractures and Fatalities of Farmyard Injuries in Ireland: A 10 Year Analysis,” Irish Medical Journal 1 (2017): 1–5. 28657272

[5] H. Young , E. Grundy , D. O'Reilly , and P. Boyle , “Self‐Rated Health and Mortality in the UK: Results From the First Comparative Analysis of the England and Wales, Scotland, and Northern Ireland Longitudinal Studies,” Population Trends 139, no. 1 (2010): 11–36. 10.1057/pt.2010.3 20379276

[6] K. S. Chan , E. Roberts , R. McCleary , C. Buttorff , and D. J. Gaskin , “Community Characteristics and Mortality: The Relative Strength of Association of Different Community Characteristics,” American Journal of Public Health 104, no. 9 (2014): 1751–1758. 25033152 10.2105/AJPH.2014.301944 PMC4151920

[7] M. Haan , G. A. Kaplan , and T. Camacho , “Poverty and Health: Prospective Evidence From the Almeda County Study,” American Journal of Epidemiology 125, no. 6 (1987): 989–998. 3578257 10.1093/oxfordjournals.aje.a114637

[8] I. H. Yen and G. A. Kaplan , “Neighbourhood Social Environment and Risk of Death: Multilevel Evidence From the Alameda County Study,” American Journal of Epidemiology 149, no. 10 (1999): 898–907. 10342798 10.1093/oxfordjournals.aje.a009733

[9] H. Som , World Programme for the Census of Agriculture 2010 (FAO Statistics Division, 2010), 1–7.

[10] B. Bunting , C. Corry , S. O' Neill , and A. Moore , “Death by Suicide at the Ward Level in Northern Ireland,” Psychological Medicine 48, no. 8 (2018): 1375–1380. 29039289 10.1017/S0033291717003026

[11] K. Steenland , J. Henley , E. Calle , and M. Thun , “Individual‐ and Area‐Level Socioeconomic Status Variables as Predictors of Mortality in a Cohort of 179,383 Persons,” American Journal of Epidemiology 159, no. 11 (2004): 1047–1056. 15155289 10.1093/aje/kwh129

[12] K. Trearty , B. Bunting , and J. Mallett , “Data Report on Three Datasets: Mortality Patterns Between Agricultural and Non‐agricultural Ward Areas,” Frontiers Statistical Genetics and Methodology 13 (2023): 1–4. 10.3389/fgene.2022.953167 PMC9851396 36685977

[13] G. Dahlgren and M. Whitehead , Policies and Strategies to Promote Social Equity in Health (Institute for the Futures Studies, 1991).

[14] K. Trearty , B. Bunting , and J. Mallett , “Assessing the Impact of Socio‐Demographics and Farming Activity on Ward‐Level Mortality Patterns Using Farm and Population Decennial Censuses,” Australian Journal of Rural Health 32, no. 2 (2024): 365–376. 38530038 10.1111/ajr.13098

[15] I. Ruiz‐Martinez , E. Marraccini , M. Debolini , and E. Bonari , “Indicators of Agricultural Intensity and Intensification: A Review of the Literature,” Italian Journal of Agronomy 10, no. 656 (2015): 74–84.

[16] D. J. Abson , E. D. Fraser , and T. G. Benton , “Landscape Diversity and the Resilience of Agricultural Returns: A Portfolio Analysis of Land‐Use Patterns and Economic Returns From Lowland Agriculture,” Agriculture & Food Security 2, no. 2 (2013): 1–15.

[17] D. Luke and M. Krauss , “Where there's Smoke there's Fire: Tobacco Industry Campaign Contributions and U.S. Congressional Voting,” American Journal of Preventitive Medicine 27, no. 5 (2004): 363–372. 10.1016/j.amepre.2004.08.014 15556735

[18] D. A. McGranahan , Natural Amenities Drive Rural Population Change (Department of Agriculture, 1999).

[19] DARD , The Agricultural Census in Northern Ireland: Results for June 2011 (Department of Agriculture Rural Development, 2012).

[20] R. MacIntosh and S. Hashim , “Variance Estimation for Converting MIMIC Model Parameters to IRT Parameters in DIF Analysis,” Applied Psychological Measurement 27, no. 5 (2003): 372–379.

[21] L. K. Muthén and B. O. Muthén , Mplus User's Guide, 8th ed. (Muthén & Muthén, 1998).

[22] A. F. Kilic , “Can Factor Scores Be Used Instead of Total Score and Ability Estimation?,” International Journal of Assessment Tools in Education 6, no. 1 (2019): 25–35.

[23] T. R. Saunderson and I. H. Langford , “A Study of the Geographical Distribution of Suicide Rates in England and Wales 1989–1992 Using Emprical Bayes Estimates,” Social Science and Medicine 43, no. 4 (1996): 489–502. 8844950 10.1016/0277-9536(95)00427-0

[24] M. Marmot , J. Allen , T. Boyce , P. Goldblatt , and J. Morrison , Health Equity in England: The Marmot Review 10 Years on (Institute of Health Equity, 2020). 10.1136/bmj.m693 32094110

[25] U. Kandler , C. Meisinger , J. Baumert , and H. Löwel , “Living Alone Is a Risk Factor for Mortality in Men but Not Women From the General Population: A Prospective Cohort Study,” BMC Public Health 7, no. 335 (2007): 1–8. 18336722 10.1186/1471-2458-7-335 PMC2225416

[26] K. Wong , A. S. Chan , and S. C. Ngan , “The Effect of Long Working Hours and Overtime on Occupational Health: A Meta‐Analysis of Evidence From 1998 to 2018,” International Journal of Environmental Research and Public Health 16 (2019): 1–22. 10.3390/ijerph16122102 PMC6617405 31200573

[27] P. M. Nilsson , S. E. Johansson , and J. Sundquist , “Low Educational Status Is a Risk Factor for Mortality Among Diabetic People,” Diabetic Medicine 15, no. 3 (1998): 213–219. 9545122 10.1002/(SICI)1096-9136(199803)15:3<213::AID-DIA569>3.0.CO;2-#

[28] D. Heenan , “Expectations and Attitudes Affecting Patterns of Informal Care in Farming Families in Northern Ireland,” Ageing and Society 20 (2000): 203–216.

[29] D. O'Reilly , S. Connolly , M. Rosato , and C. Patterson , “Is Caring Associated With an Increased Risk of Mortality? A Longitudinal Study,” Social Science and Medicine 67 (2008): 1282–1290. 18667262 10.1016/j.socscimed.2008.06.025

[30] M. T. Sánchez‐Santos , M. Mesa‐Frias , M. Choi , et al., “Area‐Level Deprivation and Overall and Cause‐Specific Mortality: 12 years' Observation on British Women and Systematic Review of Prospective Studies,” PLoS One 8, no. 9 (2013): e72656. 24086262 10.1371/journal.pone.0072656 PMC3782490

[31] J. Wakefield , “Multi‐Level Modelling, the Ecologic Fallacy, and Hybrid Study Designs,” International Journal of Epidemiology 38 (2009): 330–336. 19339258 10.1093/ije/dyp179 PMC2663723

[32] C. F. Citro , “From Multiple Modes for Surveys to Multiple Data Sources for Estimates,” Survey Methodology 40, no. 2 (2014): 137–161.

[33] G. Knies , “Exploring the Value of Understanding Society for Neighbourhood Effects Analyses,” Research Data Journal for the Humanities and Social Sciences 2 (2017): 1–22.

