# Supplementary material for: Decennial Ward‐Level Influence of Demographic, Farming, and Economic Predictors on All‐Cause Mortality
Source: Aust J Rural Health. 2025 Feb 24;33(1):e70016. doi: 10.1111/ajr.70016 (PMC11848812; doi:10.1111/ajr.70016)
Supplement: Supplementary file 1 — Appendix S1. [file AJR-33-0-s001.docx]

**Decennial Ward-Level Influence of Demographic, Farming, and**

**Economic Predictors on All-Cause Mortality**

**Appendix 1**

*NI summary statistics for mortality, sociodemographic, economic, and environmental characteristics between 2001 and 2011 censuses*

| **Variables** | **2001** | **2011** | **% Diff.** |
| --- | --- | --- | --- |
| NI Population | 1,685,267 | 1,810,863 | + 7.45 |
| Deaths | 14,513 | 14,204 | - 2.13 |
| 65 to 100+ years | 223,325 | 263,720 | +18.09 |
| Males | 821,449 | 887,323 | + 8.02 |
| Living Alone | 629,128 | 749,466 | +19.13 |
| Below Degree | 1,497,756 | 1,472,319 | - 1.70 |
| Farmers | 56,362 | 46,848 | - 16.88 |
| Full-Time hours | 533,394 | 540,224 | + 1.28 |
| Unpaid carers | 185,066 | 213,980 | +15.62 |
| LLTI | 343,107 | 374,646 | + 9.19 |
| Farms | 29,817 | 24,436 | - 18.05 |
| Grass | 993,966 | 917,986 | - 7.64 |
| Cattle | 1,679,132 | 1,590,452 | - 5.28 |
| Pigs | 385,559 | 425,268 | +10.30 |
| Poultry | 14,347,483 | 19,622,510 | +36.77 |
| Annual Farm Profit | 7,984 | 10,497 | +30.04 |

*Note. Diff.* represents the percentage difference between census years.

*LLTI* represents limiting long-term illness.

*Deprivation (NIMDM*) not included in table as it was an area ranking variable.

**Appendix 2**

*NI descriptive statistics for continuous variables with minimum, maximum, mean, standard deviation, skewness, and kurtosis characteristics in 2001 wards*

| **Variables** | **Min.** | **Max.** | **M** | **SD** | **Skew.** | **Kurt.** |
| --- | --- | --- | --- | --- | --- | --- |
| Deaths | 3.00 | 102.00 | 24.86 | 14.97 | 1.82 | 4.36 |
| Natural Log of Ward Population | 6.63 | 9.17 | 7.90 | .36 | .13 | 1.32 |
| Age | 2.93 | 28.44 | 13.59 | 4.51 | .58 | .28 |
| Males | 43.40 | 57.30 | 48.93 | 1.97 | .32 | .49 |
| Below Degree | 44.46 | 97.87 | 85.24 | 7.26 | -1.46 | 3.24 |
| Living Alone | 29.66 | 9011.00 | 48.02 | 8.89 | .94 | 1.36 |
| LLTI | 9.42 | 41.68 | 20.47 | 4.73 | .73 | .72 |
| Unpaid Care | 5.54 | 16.40 | 10.88 | 1.59 | .25 | .29 |
| Working Full-Time | 63.93 | 85.93 | 77.74 | 3.28 | -1.00 | 2.59 |
| Overall 2005 NIMDM | 1.00 | 582.00 | 291.49 | 168.16 | .00 | -1.20 |
| Average SGM Farm Profit | .00 | 155.69 | 13.72 | 16.20 | 2.30 | 12.70 |
| Farms | 0.00 | 319.00 | 51.45 | 72.11 | 1.49 | 1.30 |
| Farmers | .00 | 29.22 | 5.42 | 7.64 | 1.29 | .41 |
| Grass | .00 | 100.00 | 53.54 | 45.56 | -.29 | -1.86 |
| Total Skewness |  |  |  |  | .10 |  |
| Total Kurtosis |  |  |  |  |  | .20 |

*Note.* *NIMDM* represents Northern Ireland Multiple Deprivation Measure.

*NI descriptive statistics of livestock categorical variables and their proportion within 2001 wards*

| **Livestock** | **Categories** | | | **Wards**  **n** | **%** | **Cum. %** |
| --- | --- | --- | --- | --- | --- | --- |
| Cattle | 0 | None | 0 – 3 | 241 | 41 | 41 |
| (N = 1,679,132) | 1 | Small | 4 – 1,393 | 108 | 19 | 60 |
|  | 2 | Medium | 1,394 – 6,337 | 111 | 19 | 79 |
|  | 3 | Large | 6,338 – 16,024 | 122 | 21 | 100 |
| Pigs | 0 | None | 0 – 3 | 386 | 66 | 66 |
| (N = 385,559) | 1 | Small | 4 – 178 | 62 | 11 | 77 |
|  | 2 | Medium | 179 – 1,573 | 64 | 11 | 88 |
|  | 3 | Large | 1,574 – 17,080 | 70 | 12 | 100 |
| Poultry | 0 | None | 0 – 3 | 297 | 51 | 51 |
| (N =14,347,483) | 1 | Small | 4 – 204 | 93 | 16 | 67 |
|  | 2 | Medium | 205 – 28,631 | 93 | 16 | 83 |
|  | 3 | Large | 28,632 – 965,000 | 99 | 17 | 100 |

*Note*. 0 represents the reference category (*none* of that animal within wards).

*Wards n* represents that animal category observed within a portion of the 582 wards.

Percentages within *%* and *Cum.%* figures were rounded up.

**Appendix 3**

*NI descriptive statistics for continuous variables with minimum, maximum, mean, standard deviation, skewness, and kurtosis characteristics in 2011 wards*

| **Variables** | **Min.** | **Max.** | **M** | **SD** | **Skew.** | **Kurt.** |
| --- | --- | --- | --- | --- | --- | --- |
| Deaths | 4.00 | 100.00 | 24.41 | 13.21 | 1.69 | 4.03 |
| Natural Log of Ward Population | 6.60 | 9.16 | 7.97 | .38 | .05 | .97 |
| Age | 3.60 | 31.50 | 15.27 | 4.46 | .66 | .53 |
| Males | 43.62 | 59.78 | 49.09 | 1.71 | .54 | 2.04 |
| Below Degree | 37.30 | 94.86 | 77.48 | 8.09 | -.92 | 1.80 |
| Living Alone | 33.10 | 89.31 | 51.65 | 9.55 | .84 | .50 |
| LLTI | 9.10 | 38.40 | 21.15 | 4.69 | .66 | .35 |
| Unpaid Care | 6.89 | 16.84 | 11.78 | 1.56 | .25 | -.13 |
| Working Full-Time | 7.10 | 77.82 | 36.41 | 11.92 | .51 | .82 |
| Overall 2010 NIMDM | 1.00 | 582.00 | 291.50 | 168.15 | .00 | -1.20 |
| Average SGM Farm Profit | 3.00 | 195.81 | 19.43 | 22.77 | 2.83 | 15.24 |
| Farms | 3.00 | 272.00 | 42.95 | 60.10 | 1.55 | 1.46 |
| Farmers | .00 | 23.60 | 3.91 | 5.59 | 1.46 | 1.10 |
| Grass | .00 | 100.00 | 47.95 | 45.49 | -.06 | -1.94 |
| Total Skewness |  |  |  |  | .10 |  |
| Total Kurtosis |  |  |  |  |  | .20 |

*Note.* *NIMDM* represents Northern Ireland Multiple Deprivation Measure.

*NI descriptive statistics of livestock categorical variables and their proportion within 2011 wards*

| **Livestock** | **Categories** | | | **Wards**  **n** ^b^ | **%** ^c^ | **Cum. %** ^c^ |
| --- | --- | --- | --- | --- | --- | --- |
| Cattle | 0 | None | 0 – 3 | 272 | 47 | 47 |
| (N = 1,590,452) | 1 | Small | 4 – 2,183 | 103 | 18 | 65 |
|  | 2 | Medium | 2,184 – 6,860 | 103 | 17 | 82 |
|  | 3 | Large | 6,861 – 15,833 | 104 | 18 | 100 |
| Pigs | 0 | None | 0 – 3 | 401 | 69 | 69 |
| (N = 425,268) | 1 | Small | 4 – 105 | 60 | 10 | 79 |
|  | 2 | Medium | 106 – 1,660 | 61 | 11 | 90 |
|  | 3 | Large | 1,661 – 44,148 | 60 | 10 | 100 |
| Poultry | 0 | None | 0 – 3 | 398 | 69 | 69 |
| (N = 19,622,510) | 1 | Small | 4 – 20,697 | 61 | 10 | 79 |
|  | 2 | Medium | 20,698 – 90,200 | 61 | 10 | 89 |
|  | 3 | Large | 90,201 – 894,091 | 62 | 11 | 100 |

*Note*. 0 represents the reference category (*none* of that animal within wards).

*Wards n* represents that animal category observed within a portion of the 582 wards.

Percentages within *%* and *Cum.%* figures were rounded up.
